# Physiological and Behavioural Characterisation of a Novel Steroid Sulfatase‐Deficient Mouse

**DOI:** 10.1111/gbb.70061

**Published:** 2026-07-14

**Authors:** Trevor Humby, Freya R. Shepherd, Talia Elgie, Libby Anderson‐Watkins, Lucy I. Beevors, Angela E. Taylor, Paul A. Foster, William Davies

**Affiliations:** ^1^ School of Psychology, Cardiff University Cardiff UK; ^2^ Neuroscience and Mental Health Innovation Institute Cardiff University Cardiff UK; ^3^ Division of Psychological Medicine and Clinical Neurosciences and Centre for Neuropsychiatric Genetics and Genomics School of Medicine, Cardiff University Cardiff UK; ^4^ School of Biosciences, Cardiff University Cardiff UK; ^5^ Department of Metabolism and Systems Science College of Medicine and Health, University of Birmingham Birmingham UK

**Keywords:** atrial fibrillation, attention deficit hyperactivity disorder, dehydroepiandrosterone sulphate, Xp22.31

## Abstract

Steroid sulfatase (STS) cleaves sulphate groups from steroid hormones. In humans, STS deficiency is associated with X‐linked ichthyosis, an increased predisposition to neurodevelopmental and mood conditions (including Attention Deficit Hyperactivity Disorder, autism, depression and anxiety), and cardiac arrhythmia risk. Until recently, no single‐gene ‘knockout’ mammalian model existed; previous work in such a model is limited to skin phenotypes. We generated a novel C57BL/6J mouse model with a deletion in exon 2 of *Sts*. We examined gene expression and enzyme activity in liver and brain samples of homozygous mice, and assessed the breeding performance and health of male and female deletion‐carriers. Subsequently, we compared performance across a range of behavioural paradigms in wildtype and homozygous male and female mice: elevated plus maze, open field, rotarod, spontaneous alternation, and acoustic startle/prepulse inhibition. We also investigated serum steroid hormone levels by liquid chromatography‐mass spectrometry and measured heart weights and two morphological indices (bodyweight/tibia length) *post mortem*. Homozygous mice almost completely lacked STS expression/activity. Genetically‐altered mice exhibited grossly‐normal breeding performance, health, and endocrinology. Homozygous mice were more active and had higher normalised heart weights than wildtype mice. We also found significant genotype × sex interactions on bodyweight and on two behavioural measures (potentially reflecting lower anxiety in homozygous males and heightened anxiety in homozygous females). The ‘*Sts*‐deletion’ mouse represents an experimentally‐tractable model in which to identify and characterise phenotypes associated with STS deficiency. The mechanistic basis of the associations described here requires further investigation, and whether these translate to humans remains to be tested.

## Graphical abstract

STS‐deficient mice are grossly healthy and breed as expected, but are mildly hyperactive and have heavier hearts compared with sex‐matched wildtype controls.

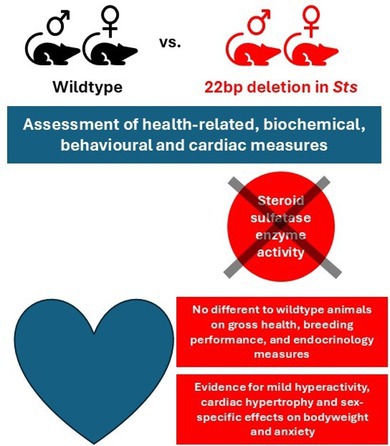

## 1 Introduction

In mammals, the enzyme steroid sulfatase (STS) is solely responsible for cleaving sulphate groups from a variety of steroid hormones (e.g., dehydroepiandrosterone sulphate, DHEAS), thereby altering their water‐solubility, activity, and availability as precursors in the biosynthesis of a number of oestrogens and androgens [1]. In humans, the *STS* gene is located at Xp22.31 and escapes X‐inactivation; hence, expression and activity levels of the enzyme are higher in females than males and might influence sex‐biased phenotypes [2]. STS is widely‐expressed, with highest levels in the placenta, arterial vasculature, adipose tissue, brain and gastrointestinal tract [3, 4].

Loss of STS function in humans, typically occurring as a consequence of either a deletion Copy Number Variant or a disruptive gene‐specific Single Nucleotide Variant (SNV), is often associated with X‐linked ichthyosis (XLI), a rare dermatological disorder characterised by skin scaling as a consequence of cholesterol sulphate accumulation in the stratum corneum [5]. Individuals affected by XLI, who are almost exclusively male, also appear at increased risk of numerous extracutaneous conditions, including cryptorchidism, corneal opacity, and fibrotic and haemostatic conditions [6]. Female carriers of XLI‐associated genetic variants may exhibit delayed or prolonged labour as a consequence of placental STS haploinsufficiency and impaired oestrogen‐mediated cervical softening, but typically present with no or mild skin abnormalities [5].

Whilst gross cognitive function and academic attainment are typically unaffected in XLI, there is some evidence for mild‐moderate impairment across aspects of fluid intelligence and memory [7, 8]. Moreover, diagnostic rates of neurodevelopmental disorders (notably Attention Deficit Hyperactivity Disorder (ADHD), dyspraxia, autism and epilepsy) in XLI are 6–8 fold higher than in the general male population, and the fact that some individuals affected by neurodevelopmental conditions possess SNVs within *STS* provides support for loss‐of‐function of this gene as being causal [6]. Rates of mood disorders and associated traits (notably those related to depression, anxiety, and irritability) in males with XLI (and female carriers) also appear significantly elevated relative to their age and sex‐matched counterparts from the general population [6, 9]. Genetic neuroimaging work has implicated smaller volume of the globus pallidus, a subregion of the basal ganglia, in vulnerability to neurodevelopmental and mood conditions in XLI [8]. We have recently shown that individuals carrying genomic deletions encompassing *STS* are at substantially increased risk of cardiac arrhythmias than non‐carriers [7]. Follow‐up genetic association analyses have implicated STS deficiency as the most likely causal mechanism [10], and we have proposed that STS deficiency confers vulnerability to structural heart abnormalities (e.g., septal defects and ventricular hypertrophy) which in turn results in increased arrhythmia risk [11]. Cardiac arrhythmias predispose to heart failure, stroke, accelerated cognitive decline/dementia, and potentially, in XLI, also to cardiac arrest [12]. These conditions, like the neurodevelopmental and mood‐related conditions mentioned above, can have long‐term effects on health and morbidity/mortality, and burden healthcare systems. Characterising behavioural and cardiac phenotypes associated with STS deficiency, and identifying underlying pathophysiological mechanisms, will be valuable for enabling earlier identification of potential issues in individuals with XLI, and for developing targeted therapeutic strategies.

Traditionally, the physiological effects of loss of a single protein during mammalian development have been investigated through the use of knockout mice; the unique highly‐repetitive genomic sequence within the mouse pseudoautosomal region has precluded such a strategy with respect to *Sts* and, as such, the impact of enzyme loss is poorly‐defined. As an alternative to the knockout mouse, the 39,X^Y^*O mouse, which lacks *Sts* as a consequence of an end‐to‐end fusion of the X and Y chromosomes has been studied. 39,X^Y^*O mice recapitulate some features of neurodevelopmental conditions including inattention, altered response inhibition, aggression, hyperactivity, increased fluid consumption, heightened emotional reactivity and perseverative behaviours, and abnormal striatal and hippocampal serotonergic function [13, 14, 15, 16, 17]. However, the 39,X^Y^*O model is necessarily male, is challenging to breed, and the deleted region includes multiple genes adjacent to *Sts* (including the autism candidate gene *Nlgn4*) [16, 18]; hence, the extent to which the aforementioned phenotypes are due to *Sts* deletion alone is unclear. The effects of selective loss of STS function in adulthood in rodents have also been investigated through acute administration of an enzyme inhibitor. These pharmacological studies have revealed effects on attention, behavioural inhibition, memory enhancement, hippocampal neurochemistry and neuroprotection in animal models relevant to dementia [13, 17, 19, 20]. Ideally, to understand the physiological role of STS, and the role of STS deficiency in the pathophysiology of XLI‐related phenotypes, we require a mammalian model in which only STS is absent throughout development, and which can be easily‐bred.

New technologies, such as CRISPR, through which a wide variety of genetic modifications can be readily made, have enabled the development of novel rodent models for understanding human disease [21]. Kwon and colleagues recently generated a mouse in which part of *Sts* exon 2 (encoding the enzyme's sulfatase domain [22]) was deleted; their characterisation of skin morphology and biochemistry in this model implicated excessive generation of reactive oxygen species, dysregulation of Wnt/β and Hippo signalling pathways (implicated in regulation of organ size through effects on cell proliferation and apoptosis), and over‐expression of E‐cadherin as mechanisms contributing to XLI skin phenotypes [23, 24]. Here, we describe the generation, and initial physiological and behavioural characterisation, of an independent novel CRISPR‐generated mouse model harbouring a small frameshift deletion in *Sts* exon 2, predicted to result in a premature stop codon. Guided by previous work in 39,X^Y^*O and STS‐inhibition models, we initially focussed on basic sensory, motoric and exploratory paradigms. We also analysed serum steroid levels and normalised heart weights (a simple index of cardiac hypertrophy) in our new model. Given the early and exploratory nature of these investigations in this new model, a pre‐registered protocol was not prepared.

## 2 Materials and Methods

### 2.1 Generation, Genotyping, Breeding and Husbandry of Mice

C57BL/6J‐Sts^em2H^/H mice were obtained from the Mary Lyon Centre at MRC Harwell which is the UK node of the European Mouse Mutant Archive (EMMA) (www.infrafrontier.eu; repository number EM:15127). Mice were generated in collaboration with us as part of the Genome Editing Mice for Medicine (GEMM) programme according to the protocol in [25], and specific details are provided in Supporting Information. Wildtype, heterozygous, and homozygous adult male and female mice were transported to Cardiff University, and initial breeding was conducted to generate heterozygous parents. Subsequently, heterozygous × heterozygous crosses were used to generate male and female wildtype, heterozygous and homozygous littermates. Mice were genotyped by Transnetyx (Cordova, TN) from genomic DNA obtained from ear biopsies at weaning using real‐time PCR probes specific for wild type and mutant alleles; eight mice were re‐genotyped *post mortem* and results were identical to previous calls. Mice were housed in same‐sex mixed‐genotype groups (2–5 animals per cage), and were maintained on ad libitum food and water, in a temperature, humidity and light‐controlled room (21°C ± 2°C, 50% ± 10% humidity, lights on at 0800 h for 12 h) with environmental enrichment, and were regularly inspected for signs of ill health. Animals of each genotype for the various analyses came from ≥ 6 homecages and individual animals were the experimental unit. Unless otherwise stated, researchers were aware of animals' genotypes prior to testing; most measures were obtained automatically, or were blindly double‐scored, to minimise potential researcher bias. Animals of the various genotypes were tested in a randomised order based upon their number assigned at weaning (i.e., prior to genotypes being known). Raw data for wildtype and homozygous animals are available in Supporting Information S1.

### 2.2 Behavioural Analysis

92 adult (3–7 months) mice (wildtype (+/+) males (*n* = 12), heterozygous (+/−) males (*n* = 15), homozygous (−/−) males (*n* = 22), wildtype females (*n* = 14), heterozygous females (*n* = 15), and homozygous females (*n* = 14)) underwent behavioural testing conducted by a female researcher during the light phase (0900–1500 h). For the sake of clarity the heterozygote data are not reported here, but across the vast majority of measures, these groups presented, as expected, intermediately between wildtype and homozygote groups; behavioural data for these animals are available in Supporting Information S2. This sample size allowed medium‐large effect sizes (*f* > 0.36) between wildtype and homozygous animals to be reliably detected (with > 80% power, *α* = 0.05); such effect sizes are comparable with those we have seen previously when comparing 40,XY and 39,X^Y^*O mice [14].

Following 2 weeks' habituation to handling, a series of behavioural tests assaying anxiety‐related behaviours, locomotor activity, motoric function/co‐ordination, exploratory behaviours and startle response/sensorimotor gating was conducted across 3 days, with the least stressful tests taking place first and with at least 90 min between tests to allow stress responses to return to baseline. On each test day, male mice were run before female mice, with animals run in a pseudorandomised order with respect to genotype. Test apparatus was cleaned with 70% industrial methylated spirits between animals to disinfect it and to limit any potential confounding effects of odour. Mice were allowed to habituate to each testing room for ≥ 30 min prior to testing. The order of testing was: elevated plus maze (EPM), open field test (OFT), assessment of consummatory behaviour, rotarod, spontaneous alternation and finally the assessment of acoustic startle response and sensorimotor gating.

The EPM and OFT were run under identical lighting conditions (15 lx) and the position of each mouse in the apparatus was automatically tracked using EthoVision XT software (Noldus Information Technology, Netherlands) at a rate of 12 frames/s via a camera mounted above the centre of each piece of apparatus. The EPM was constructed of white Perspex and consisted of four arms of equal size (175 × 78mm) extending from a central square region (78 × 78mm) and positioned 450 mm above the floor. Two of the arms were ‘open’ (no walls) and two were enclosed by 150 mm high opaque walls (‘closed’). Arms of the same type were diametrically opposed. Animals were placed in the same enclosed arm, facing the wall, at the start of the trial, and were allowed to freely explore the apparatus for 5 min. Key EPM outcome measures assaying anxiety‐related behaviours and/or within‐maze activity included: the ratio of time spent in the open and closed arms (×100), total distance moved, latency to enter the open arm and number of faecal boli deposited. The OFT comprised a 750 × 750mm white Perspex arena with 450 mm high walls. Animals were placed in the same corner of the arena, facing the wall, and were allowed to explore freely for 10 min. The arena was divided into two concentric virtual zones: the ‘inner zone’ (central 600x600mm square) and the ‘outer zone’. Key OFT outcome measures assaying anxiety‐related behaviours and/or within‐maze activity included: time in the inner zone, total distance moved, latency to enter the inner zone, and number of faecal boli deposited.

To assess baseline drinking behaviour and response to a novel foodstuff, mice with previous *ad libitum* access to homecage water were individually placed into a small chamber (285 × 130 × 120mm, length × width × height) for 10mins in which two small containers (maximum volume ~3 mL each) were positioned towards the rear, one containing tap water and the other a 10% condensed milk solution (Nestle Ltd) novel to the mouse. Containers were weighed at the start and end of the session and the total fluid volume consumed by each animal calculated; this figure was then normalised to bodyweight^0.75^ to take into account Kleiber's 0.75 mass exponent and multiplied by 100. The volume of milk consumed as a percentage of total fluid consumed was also calculated.

Motor learning and co‐ordination were assessed using a mouse rotarod (Model 47600, Ugo Basile, Italy). The rod (30 mm diameter) was coated with rubber grooves to provide grip. Each animal completed five trials of 300 s maximum duration with ≥ 30 min recovery time between each trial. During each trial, the rotation speed increased from 5 to 50 rpm at a constant rate of 0.15 rpm/s. The latency to fall was recorded on each trial, when the mouse caused a timer to stop when triggered by a lever (160 mm below the rod). For each animal, the median time spent on the apparatus before falling across the five trials was determined.

Exploratory and spontaneous alternation behaviour, partially indexing working memory ability, was assayed by testing animals in a T‐maze made of clear Perspex with a ‘start arm’ 54.5 cm long and 10 cm wide, two ‘goal arms’ 25.5 cm long and 10 cm wide, a ‘decision’ space 10 cm × 10 cm, and walls 25 cm high. Mice were initially placed on the floor at the end of the ‘start’ arm and were allowed to explore freely for 10 min with their behaviour being video‐recorded. To enhance between‐arm discriminability, the right arm was covered in a checkerboard pattern (alternating squares 2.5 × 2.5 cm) and the left arm covered in a dot pattern (black dots of 1 cm diameter). Key outcome measures included: total arm entries (a measure of locomotor activity defined by all four paws in an arm) and total alternations (defined by consecutive visits to the three different arms). These measures were initially scored from videos by an experienced analyst unaware of animals' genotype and sex, and were double‐scored by a second blinded researcher. The ratio of spontaneous alternations to maximum possible spontaneous alternations (i.e., total arm entries‐2) was then calculated. Inter‐rater reliability for the key measures was determined via calculation of the intra‐class correlation coefficient (ICC).

Acoustic startle response (ASR) and prepulse inhibition (PPI) of this, indices of reactivity to an unpredictable auditory stimulus and sensorimotor gating respectively, were measured using apparatus from SR‐Lab (San Diego Instruments, USA). Animals were placed in a clear Perspex tube (35 mm internal diameter) mounted on a Perspex plinth in a sound‐attenuating chamber. A 70 dB background white noise stimulus was continuously played throughout the session via a loudspeaker positioned 120 mm above the tube. The whole‐body startle response was detected on each trial by a piezoelectric sensor attached to the plinth, which transduced flexion in the plinth into a digitised signal. The session comprised 44 trials in total, following a 5 min habituation period with background sound and lasted for approximately 30 min. The startle response was recorded in arbitrary startle units using SR‐Lab software over the 65 ms period following the main startle stimulus onset. Acoustic stimuli were presented with a mean intertrial interval of 16 s (pseudorandomly varied between 6 and 24 s). Each pulse‐alone trial consisted of a 40 ms 120 dB startle stimulus. The median startle response for each mouse across the final 8 pulse‐alone trials was calculated and the resultant figure divided by bodyweight and multiplied by 10. Prepulse trials consisted of a 20 ms prepulse stimulus (4, 8, or 16 dB) followed by a 40 ms 120 dB startle stimulus occurring 80 ms after prepulse offset. There were six trials at each prepulse amplitude randomly interspersed amongst the pulse‐alone trials. PPI was calculated as the percentage reduction in mean startle amplitude between prepulse and pulse‐alone trials.

### 2.3 Tissue Collection

80 behaviourally‐tested, and behaviourally‐naïve, adult (3–11.5 months) mice were weighed and culled by cervical dislocation between 09:00–15:00 h, with the culling order pseudorandomised by genotype (wildtype males (*n* = 15), homozygous males (*n* = 29), wildtype females (*n* = 21), homozygous females (*n* = 15)). Trunk blood was collected and serum extracted using BD Microtainer SST tubes (Fisher Scientific) for steroid analysis. Whole brain and liver samples were also taken for enzyme activity and gene expression analyses (*n* = 2 per group). Serum, brain and liver samples were snap‐frozen on dry ice and stored at −80°C. Hearts from older animals (> 20 weeks) were dissected, residual blood removed, and their wet weight recorded; tibias from these animals were also dissected, and their length measured within 0.1 mm using digital vernier callipers. For each animal, heart weight was divided by bodyweight and tibia length, and the resultant figure multiplied by 1000. For the bodyweight and heart weight measures, the study was powered to reliably detect (> 80% power) a genotype‐dependent medium effect size (*f* > 0.35, *α* = 0.05).

### 2.4 Enzyme Activity and Gene Expression Analyses

STS activity in liver and whole brain tissues was assayed by following our previously‐published cell‐free method with minor modifications for solid samples [26]; details are provided in Supporting Information. Individual animals' samples were assayed in triplicate and the median value taken for statistical analysis. Additionally, *Sts* gene expression in liver samples from homozygous and wildtype mice was assayed using quantitative PCR (methods in Supporting Information).

### 2.5 Serum Steroid Analysis

Serum steroid levels were quantified at the Steroid Metabolome Analysis Core, University of Birmingham, using a validated assay described previously [27]; details are provided in Supporting Information.

### 2.6 Statistical Analysis

Statistical analysis was performed with IBM SPSS Statistics 27 software. Categorical breeding data were analysed by chi‐squared test. Following removal of group outliers (< 1.5 times the interquartile range below the 25th percentile or above the 75th percentile, < 3.5% of all datapoints), most behavioural and cardiac morphology data were analysed by Two Way ANCOVA with factors of GENOTYPE (wildtype or homozygote) and SEX (male or female), and age as a covariate; estimated marginal means are reported for significant interaction effects. Analysis of the prepulse inhibition data also included the repeated measures factor of PREPULSE AMPLITUDE (4, 8 or 16 dB) with Greenhouse–Geisser correction to adjust for lack of sphericity. Where assumptions underlying ANCOVA were violated (non‐normal distribution of residuals in ≥ 2 groups assessed by Shapiro–Wilk test, or non‐homogeneity of variance assessed by Levene's test), data were appropriately transformed as far as possible (natural log or reciprocal). For analyses where ANCOVA assumptions were significantly violated (e.g., for steroid hormone analysis), comparisons were conducted by Mann–Whitney *U*‐test or Kruskal–Wallis *H*‐test with follow‐up Bonferroni‐corrected pairwise comparisons to adjust for multiple testing. *p*‐values < 0.05 were regarded as being statistically‐significant. Data are shown as box and whisker plots where centre lines indicate median values, box limits indicate the 25th and 75th percentiles and whiskers extend 1.5 times the interquartile range from the 25th and 75th percentiles; outlying datapoints reside outside the plot whiskers.

## 3 Results

### 3.1 Effect of Genetic Manipulation on STS Enzyme Activity and Gene Expression

Consistent with previous mouse data [28], STS activity was higher in wildtype liver tissue (313.3 ± 105.3 pmol/mg/h, *n* = 2 per sex) than in wildtype brain tissue (53.2 ± 9.4 pmol/mg/h, *n* = 2 per sex). As expected, the genetic deletion resulted in substantial attenuation of STS activity in homozygous mice across both tissues. In liver, STS activity was barely detectable in homozygous animals (*n* = 2 per sex) at 1.83 ± 1.83 pmol/mg/h (> 99% reduction compared to wildtype animals, *U* = 16.0, *p* = 0.03). In brain, STS activity in homozygote animals was just 5.6 ± 4.1 pmol/mg/h (> 85% reduction compared to wildtype animals, *U* = 16.0, *p* = 0.03). These enzyme activity data mirrored our gene expression data, where *Sts* gene expression in homozygous mouse liver tissue was markedly lower (~15‐fold) than that in wildtype tissue (Figure S1).

### 3.2 General Health, Bodyweight and Breeding Performance

**FIGURE 1:**
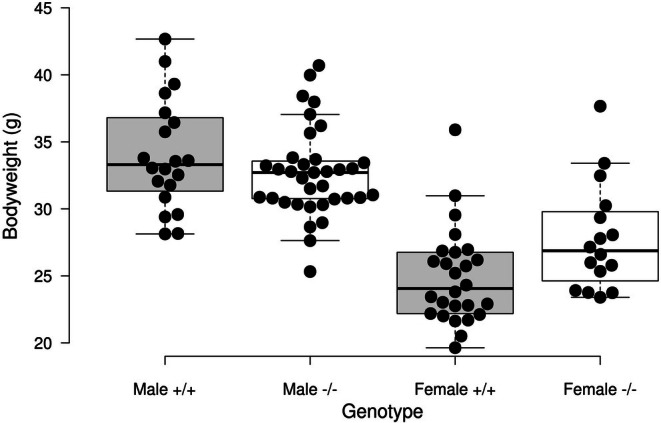
Bodyweight at culling in adult male and female wildtype (+/+) and homozygous (−/−) mice.

The vast majority (> 95%) of male and female heterozygous and homozygous mice exhibited normal health from birth to the latest timepoint assessed (12 months). Of the 300 adult genetically‐altered mice (heterozygotes or homozygotes) generated in our work to date, three weaned heterozygous mice (two male, one female) were found dead of an indeterminate cause (1.0%) and one female homozygous mouse required culling due to severe illness of uncertain origin (0.3%). Two heterozygous and one homozygous adult female mice were identified as having vaginal septa (2.1% of genetically‐altered females), and of the 65 genetically‐altered female mice used for breeding to date, four heterozygotes (6.2%) exhibited evidence of dystocia. Measurement of bodyweight across animals aged 98–348 days revealed no significant age‐adjusted main effect of GENOTYPE (*F*[1,85] = 0.23, *p* = 0.63); however, there was the expected significant effect of SEX (males > females) (*F*[1,85] = 259.7, *p* < 0.001) and an unexpected significant GENOTYPE × SEX interaction, whereby homozygous males were ~5% lighter (estimated marginal mean = 32.5 ± 0.4 g), and homozygous females ~5% heavier (estimated marginal mean = 25.8 ± 0.6 g), than their sex‐matched wildtype littermates (estimated marginal means of 34.0 ± 0.5 g and 24.7 ± 0.5 g respectively) (*F*[1,85] = 6.20, *p* = 0.02) (Figure 1).

To date in our breeding colony, 303 mice from 54 litters have survived to weaning from heterozygote × heterozygote crosses (median litter size of six pups). At weaning, the genotype ratios obtained were consistent with the expected Mendelian ratios: wildtype male (*n* = 31, 10%), heterozygote male (*n* = 93, 31%), homozygote male (*n* = 45, 15%), wildtype female (*n* = 33, 11%), heterozygote female (*n* = 73, 24%) and homozygote female (*n* = 28, 9%) (*χ* ^2^[5] = 9.8, *p* = 0.08). The genotypes of 26 unsexed pups found dead between birth and weaning were also in the expected ratios wildtype (*n* = 4, 15%), heterozygote (*n* = 14, 54%), homozygote (*n* = 8, 31%) (*χ* ^2^[2] = 1.38, *p* = 0.50), suggesting no large effect of genotype on deaths in this period.

### 3.3 Behavioural Analysis

#### 3.3.1 Elevated Plus Maze Test

**FIGURE 2:**
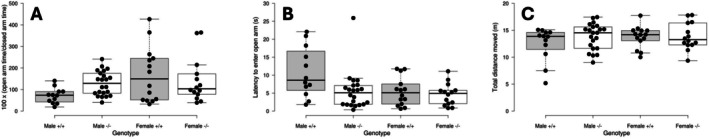
Performance of adult male and female wildtype (+/+) and homozygous (−/−) mice on key measures of anxiety‐related behaviour (open:Closed arm time (A) and latency to enter open arm (B)) and activity (total distance travelled) on the elevated plus maze test.

On the main measure of anxiety‐related behaviour in this task (ratio of time spent on the open arms relative to time spent in the enclosed arms), there was no main effect of GENOTYPE (*F*[1,55] = 0.44, *p* = 0.51), but there was a significant main effect of SEX, whereby females tended to spend more time on the open arms relative to males (*F*[1,55] = 4.70, *p* = 0.03)(Figure 2A). There was also a GENOTYPE × SEX effect on this measure, with homozygous males tending to spend more time on the open arms (estimated marginal mean = 132.6 ± 13.7), and homozygous females tending to spend less time on the open arms (estimated marginal mean = 115.5 ± 18.6), than their wildtype sex‐matched controls (estimated marginal means of 67.2 ± 18.5 and 158.1 ± 17.2 respectively) (*F*[1,55] = 10.0, *p* = 0.003)(Figure 2A). On the ‘latency to enter the open arm’ measure (Figure 2B), there was a significant main effect of GENOTYPE, with homozygous animals tending to enter the open arms sooner in the test than wildtype mice (*F*[1,56] = 4.23, *p* = 0.04); the main effect of SEX on this measure was non‐significant (*F*[1,56] = 3.32, *p* = 0.07) as was the GENOTYPE × SEX interaction (*F*[1,56] = 3.25, *p* = 0.08). There were no significant group effects on a final measure of anxiety‐related behaviour in this test: number of faecal boli deposited (*H*[3]=0.35, *p* = 0.95). The significant effects on anxiety‐related behaviour described above occurred in the absence of significant effects of GENOTYPE (*F*[1,55] = 0.13, *p* = 0.72), SEX (*F*[1,55] = 0.74, *p* = 0.39), or GENOTYPE × SEX (F[1,55] = 0.58, *p* = 0.45) on activity (total distance moved within the maze) (Figure 2C). Covarying for ‘distance moved’ did not impact the pattern of results with respect to open: closed arm time, but resulted in attenuation of the genotype‐dependent effect on ‘open arm entry latency’ (*F*[1,55] = 3.95, *p* = 0.052) implying its partial dependence upon general activity levels.

#### 3.3.2 Open Field Test

**FIGURE 3:**
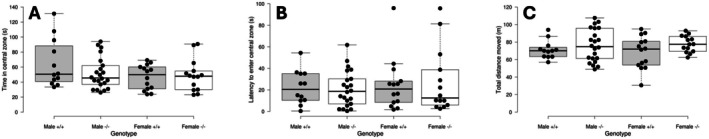
Performance of adult male and female wildtype (+/+) and homozygous (−/−) mice on key measures of anxiety‐related behaviour (time in central zone (A) and latency to enter central zone (B)) and activity (total distance travelled (C)) on the open field test.

We observed no significant effects of GENOTYPE (*F*[1,57] = 1.14, *p* = 0.29), SEX (*F*[1,57] = 2.77, *p* = 0.10) or GENOTYPE × SEX (*F*[1,57] = 1.74, *p* = 0.19) on the main measure of anxiety‐related behaviour (time spent in the central zone) (Figure 3A). A similar null pattern of findings was observed with respect to a second measure of anxiety‐related behaviour (latency to first enter the central zone): effect of GENOTYPE (*F*[1,55] = 0.19, *p* = 0.67), effect of SEX (*F*[1,55] = 0.10, *p* = 0.75) and GENOTYPE × SEX (*F*[1,55] = 0.33, *p* = 0.57) (Figure 3B). There were also no significant group effects on a third measure of anxiety‐related behaviour in this test: number of faecal boli (*H*[3]=1.44, *p* = 0.70). We did, however, identify a genotype‐dependent effect on locomotor activity (total distance moved) in this relatively non‐aversive, unrestricted test (*F*[1,56] = 4.19, *p* = 0.045) with homozygotes being ~10% more active than their wildtype counterparts. There was no significant main effect of SEX (*F*[1,56] = 0.004, *p* = 0.95), nor a significant GENOTYPE × SEX interaction (*F*[1,56] = 0.13, *p* = 0.72) with respect to open field activity (Figure 3C).

#### 3.3.3 Baseline Drinking Behaviour and Reactivity to a Novel Foodstuff

There was a significant overall group effect on total fluid volume consumed normalised to bodyweight^0.75^ (*H*[3]=8.51, *p* = 0.04). This effect appeared due to a combination of higher consumption in homozygous male mice relative to wildtype male mice (resembling the effect reported in 39,X^Y^*O mice previously [14]) and slightly lower consumption in homozygous female mice relative to wildtype female mice; however, no individual adjusted pairwise comparisons were significant (Figure S2A). With respect to solution preference (water vs. milk), there was no significant effect of GENOTYPE (*F*[1,57] = 0.20, *p* = 0.65) or SEX (*F*[1,57] = 2.44, *p* = 0.12), but there was a significant GENOTYPE × SEX interaction (*F*[1,57] = 5.69, *p* = 0.02) mirroring the baseline drinking pattern, with homozygous males exhibiting a greater preference for the milk solution (estimated marginal mea*n* = 61.4 ± 4.4) than sex‐matched wildtype mice (estimated marginal mean = 50.9 ± 6.0), and homozygous females exhibiting a reduced preference for the milk solution (estimated marginal mean = 40.0 ± 5.6) relative to sex‐matched wildtype mice (estimated marginal mean = 55.4 ± 5.6) (Figure S2B). Across the four groups, there was no significant group effect on number of faecal boli (H[3]=3.13, *p* = 0.37).

#### 3.3.4 Rotarod Test

Median time on the rotarod across the five trials was equivalent across the two genotypes (*F*[1,57] = 0.94, *p* = 0.34) and the two sexes (*F*[1,57] = 0.33, *p* = 0.57), and there was no significant GENOTYPE × SEX interaction (*F*[1,57] = 3.49, *p* = 0.07) (Figure S3). The number of faecal boli deposited on the final trial did not differ by group (*H*[3]=2.15, *p* = 0.54).

#### 3.3.5 Spontaneous Alternation Test

The behavioural performance of a subset of 30 animals split across genotypes and sexes was independently, and blindly, double‐scored for key measures. There was very good–excellent, and highly significant (*p* < 0.001), agreement between blinded raters: intra‐class correlation coefficients = 0.97 for ‘total arm entries’, 0.87 for ‘spontaneous alternations’, and 0.81 for ‘spontaneous alternations as a function of maximum possible alternations’.

**FIGURE 4:**
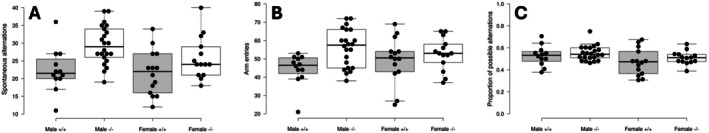
Performance of adult male and female wildtype (+/+) and homozygous (−/−) mice on alternation (A), activity (B), and alternations normalised for activity (C) on the spontaneous alternation test.

There was a highly‐significant effect of GENOTYPE on the total number of spontaneous alternations (*F*[1,55] = 15.29, *p* < 0.001) reflecting higher numbers of alternations in homozygous mice, but no effect of SEX (*F*[1,55] = 1.94, *p* = 0.17) and no GENOTYPE × SEX interaction (*F*[1,55] = 1.13, *p* = 0.29) (Figure 4A). Similarly, there was a significant effect of GENOTYPE on total arm entries (*F*[1,55] = 4.56, *p* = 0.04) consistent with more arm entries in homozygous mice, but there was no significant effect of SEX (*F*[1,55] = 0.14, *p* = 0.71) or GENOTYPE × SEX (*F*[1,55] = 2.62, *p* = 0.11) observed (Figure 4B). There was no significant overall group effect on the proportion of alternations as a function of the number of maximum possible alternations (*H*[3]=6.11, *p* = 0.11) (Figure 4C). There was also no overall group effect on the number of faecal boli deposited (*H*[3]=5.05, *p* = 0.17).

#### 3.3.6 Acoustic Startle Response and Prepulse Inhibition

There was no significant effect of GENOTYPE (*F*[1,52] = 1.22, *p* = 0.28), SEX (*F*[1,52] = 1.15, *p* = 0.29) or GENOTYPE × SEX (*F*[1,52] = 1.69, *p* = 0.20) on the basic startle response normalised for bodyweight (Figure S4A). Although the expected increase in startle inhibition was seen with increasing PREPULSE AMPLITUDE across groups (*F*[1.51,87.7] = 93.3, *p* < 0.001), there was no significant effect of GENOTYPE (*F*[1,57] = 2.06, *p* = 0.16), SEX (*F*[1,57] = 0.53, *p* = 0.47), or GENOTYPE × SEX interaction (*F*[1,57] = 0.28, *p* = 0.60) on prepulse inhibition (Figure S4B).

### 3.4 Steroid Hormone Analysis

21 steroids in total were assayed in serum across four groups: wildtype males (*n* = 6), wildtype females (*n* = 5), homozygous males (*n* = 15), and homozygous females (*n* = 3). The four groups did not differ significantly by age (*H*[3]=4.21, *p* = 0.24). Eight steroids (pregnenolone, 17α‐hydroxyprogesterone, 11‐deoxycorticosterone, androstenedione, 11‐ketoandrostenedione, 11‐ketotestosterone, 11β‐hydroxyandrostenedione, 11β‐hydroxytestosterone) were not detectable in > 10% of all samples, with lack of detectability not differing significantly by group for seven of these (0.13 < *p* < 1.0); androstenedione was less frequently detectable in female than male samples (~33% vs. ~80%, *p* = 0.04). Levels of the remaining 13 steroids were undetectable in < 1.5% of all sample readings, where they were assigned a zero value. For testosterone, dihydroepiandrosterone, and 5α‐androstanedione, there were significant overall and pairwise effects across the four groups, consistent with the expected higher levels in male compared to female groups, but not indicative of genotype‐dependent effects (Table 1). For aldosterone, there was a significant effect of group, but no significant pairwise comparisons. For the other readily‐detectable compounds assayed, there was no evidence for either genotype or sex‐dependent effects.

**TABLE 1:** Median concentrations (ng/mL) of serum steroid hormones across groups with 95% confidence intervals defined by bootstrapping.

Steroid	Wildtype male (*n* = 6)	Wildtype female (*n* = 5)	Homozygote male (*n* = 15)	Homozygote female (*n* = 3)	H[3], *p*‐value
Progesterone	0.54 (0.11–0.70)	0.87 (0.44–2.39)	0.60 (0.29–1.02)	0.75 (0.32–2.68)	3.13, 0.37
Deoxycorticosterone	0.71 (0.11–1.26)	0.53 (0.25–11.26)	0.73 (0.38–1.25)	1.75 (0.36–3.10)	1.30, 0.73
Dehydrocorticosterone	42.42 (7.30–53.15)	50.38 (15.72–141.83)	49.74 (28.97–79.69)	85.22 (19.04–92.21)	1.82, 0.61
Corticosterone	51.04 (28.66–114.87)	100.74 (61.21–736.95)	107.64 (52.29–176.58)	77.51 (52.48–255.07)	3.08, 0.38
Aldosterone	0.11 (0.02–0.25)	0.15 (0.04–0.52)	0.25 (0.18–0.35)	0.10 (0.09–0.16)	8.91, 0.03*
5α‐androstanedione	1.24 (0.73–5.90)	0.04 (0.014–0.12)^a^	4.02 (2.47–6.03)^a^	0.05 (0.02–0.10)	14.37, 0.002*
Allopregnanolone	1.29 (0.82–2.79)	1.07 (0.68–1.46)	3.15 (1.31–4.19)	0.97 (0.34–1.40)	5.73, 0.13
Dehydroepiandrosterone (DHEA)	3.92 (2.32–8.87)	5.81 (3.49–15.10)	4.52 (2.37–8.80)	9.18 (3.71–16.54)	2.41, 0.49
Testosterone	1.56 (0.38–12.99)^c^	0.01 (0.00–0.11)^b,c^	2.35 (0.29–5.30)^a,b^	0.01 (0.00–0.01)^a^	16.85, < 0.001*
5α‐17hydroxyprogesterone	2.29 (1.74–3.05)	2.14 (1.67–3.16)	2.06 (1.74–2.51)	2.21 (1.14–4.62)	0.56, 0.91
Androsterone	13.11 (6.00–16.79)	14.93 (12.54–19.92)	13.57 (12.77–17.64)	9.87 (8.14–27.89)	1.84, 0.61
Dihydroepiandrosterone	0.05 (0.04–0.24)	0.02 (0.00–0.02)^a^	0.18 (0.03–0.26)^a^	0.01 (0.00–0.08)	11.31, 0.01*
Androst‐5‐ene‐7,17‐dione	1.50 (0.29–5.25)	2.04 (0.00–18.80)	0.73 (0.07–3.34)	5.39 (0.99–30.11)	2.09, 0.55

*Note:* Asterisks indicate significant (*p* < 0.05) overall group effects, and subscript letters a–c denote significant (*p* < 0.05) pairwise comparisons.

#### 3.4.1 Normalised Heart Weights

**FIGURE 5:**
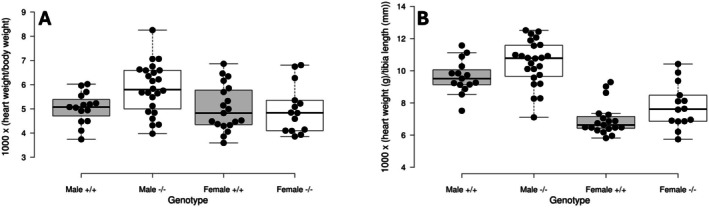
Heart weight relative to two morphological features (bodyweight (A) and tibia length (B)) in older (> 20 weeks) adult male and female wildtype (+/+) and homozygous (−/−) mice.

In older mice (> 20 weeks), where heart pathology would be expected to be greatest, there was a significant age‐adjusted effect of GENOTYPE on heart weight: bodyweight (*F*[1,67] = 4.91, *p* = 0.03), but there was no significant effect of SEX (*F*[1,67] = 2.72, *p* = 0.10) nor any significant GENOTYPE × SEX interaction (*F*[1,67] = 2.23, *p* = 0.14) (Figure 5A). With respect to the heart weight: tibia length measure (Figure 5B), after covarying for age, there was a highly significant effect of GENOTYPE (*F*[1,62] = 10.78, *p* = 0.002, reflecting a ~10%–20% greater ratio in homozygous animals compared to wildtype animals), as well as a highly significant effect of SEX (males > females) (*F*[1,62] = 109.15, *p* < 0.001), consistent with previous data on this measure in C57BL/6J mice [29]. There was no significant GENOTYPE × SEX interaction on the heart weight: tibia length ratio (*F*[1,62] = 0.13, *p* = 0.72).

## 4 Discussion

The steroid sulfatase enzyme, encoded by the sex‐linked *STS* gene, catalyses the conversion of sulphated to free steroids. In humans, STS deficiency is associated with a range of physiological and behavioural phenotypes, including X‐linked ichthyosis. Although skin phenotypes have been investigated previously in mice with a null mutation within *Sts*, the consequences of STS deficiency on general health, physiology and behaviour in mice have not yet been described.

We generated a novel genetic mouse model in which significantly attenuated STS expression and activity were predicted. We confirmed that this was the case in central (brain) and peripheral (liver) tissues of homozygous mice, although there was some degree of residual STS activity in the former; the efficiency of murine nonsense‐mediated decay processes can differ by tissue [30].

Heterozygous females gave birth to litters of equivalent size to those seen in wildtype mice of the C57BL/6 background strain [31] and in the expected genotype ratios, and mice lacking STS activity exhibited grossly normal health until at least 12 months of age. These data suggest that STS deficiency in mice, as in humans, has no major effects on general health or mortality. Reproductive health issues (vaginal septa and dystocia) were noted in genetically‐altered female mice. The prevalence of the former issue was comparable to that seen in wildtype mice of the same strain (< 5%) [32], and, as such does not appear to be genotype‐dependent. The prevalence of dystocia in (functionally) wildtype C57BL/6 mouse mothers is ~0%–8% [33, 34, 35], and in wildtype members of other multiparous small mammalian species is ~5%–8% [36]; the prevalence we observed in heterozygous mice (~6%) is within this normal range. However, given that heterozygosity for *STS*‐null variants is associated with delayed or prolonged labour in humans, this phenotype should remain an area of focus as more data are collected from further breeding pairs. To minimise any potential welfare issues, younger heterozygous mice (< 5 months of age) should be used for breeding.

Our preliminary finding of lower bodyweight in STS‐deficient males compared to sex‐matched wildtype controls, but higher bodyweight in STS‐deficient females compared to sex‐matched wildtype controls is interesting and worthy of follow‐up. Should it be replicated in additional experimental cohorts, its mechanistic basis might be investigated further, for example, examining whether the differences arise due to genotype‐dependent effects on muscle mass, levels of adiposity, food intake, metabolism and/or activity levels. To date, no systematic studies have been conducted examining bodyweight in humans lacking STS; if our findings are robust and translatable, we might expect males diagnosed with XLI to be lighter than their unaffected peers, and female carriers to be heavier than equivalent non‐carriers, and for the mechanistic basis of any phenotypes to be conserved across species.

Behaviourally, the most consistent positive finding was a small but significant genotype‐dependent effect on general activity measures across two tests (distance travelled in the open field and number of arm entries in the spontaneous alternation paradigm); elevated activity levels as a consequence of STS deficiency may contribute to the hyperactivity phenotype described previously in 39,X^Y^*O mice [14]. This finding supports the idea that STS deficiency in humans predisposes to increased hyperactive–impulsive traits and ADHD vulnerability [37, 38], although activity levels in Xp22.31 deletion carriers have yet to be explicitly tested (e.g., using actigraphy). Future mouse work might examine the neurobiological basis of this hyperactivity phenotype and, in particular, test whether it can be alleviated with medications used clinically to treat ADHD.

We found no significant main effects of genotype on any of the multiple anxiety‐related behavioural measures we assessed. However, we did observe subtle, activity‐independent, genotype by sex interactions for two such measures: open: closed arm time in the elevated plus maze, and willingness to consume a novel, and potentially toxic, solution. In both cases, homozygous male mice displayed behaviours consistent with lower anxiety (or greater risk‐taking) than wildtype males (i.e., spending relatively more time on the open arms, and greater consumption of the novel foodstuff), whereas homozygous female mice displayed behaviours consistent with greater anxiety (or lower risk‐taking) than wildtype females (i.e., spending relatively less time on the open arms, and lower consumption of the novel foodstuff). Interestingly, amongst hyperactive individuals diagnosed with ADHD, the sexes display differences related to risk‐taking behaviours and anxiety [39, 40]. Although the present findings could genuinely index effects on specific aspects of anxiety, it should be acknowledged that they could be Type I errors given our low power to reliably detect interactions, and the milk consumption data likely reflects hedonic (as well as anxiety‐related or impulsivity‐related) responses.

Our mouse findings contrast somewhat with findings in individuals with XLI, or female carriers, where elevated levels of generalised anxiety‐related traits and anxiety disorder diagnoses are apparent [9]. Perhaps the anxiety‐related effects in man are due to loss of Xp22.31 genes other than *STS*, or perhaps STS has species‐specific effects on anxiety processes. Testing individuals with XLI, or female carriers, in experimental paradigms more closely‐related to those giving rise to significant findings in mice (e.g., virtual elevated plus maze [41]), might reveal analogous results.

For the remaining behavioural measures, indexing gross motor coordination (rotarod performance), reactivity to an acoustic stimulus and sensorimotor gating (startle response and inhibition of this upon exposure to a prepulse), and exploratory behaviour and working memory (spontaneous alternation), we observed no significant effects of genotype, or genotype interactions with sex. The fact that STS‐deficient mice perform equivalently to wildtype mice in terms of these basic behaviours, means that they can be used subsequently without major issues for more sophisticated cognitive testing (e.g., assaying measure of attention and impulsivity associated with ADHD); however, the positive findings above (e.g., hyperactivity) will need to be accounted for in subsequent work.

Our endocrinological analysis identified the expected large sex differences in levels of testosterone and 5α‐androstanedione, indicating that any large genotype‐dependent effects on serum hormones should have been detectable. If genotype differences in serum levels of the hormones we assayed here do genuinely exist, they are likely to be moderate or small in magnitude. Although we found an overall significant effect of group on circulating aldosterone levels, there were no significant *post hoc* pairwise comparisons. While this significant result is probably a Type I error due to limited sample sizes per group, it is noteworthy that aldosterone levels are associated with cardiovascular outcomes including arrhythmia and hypertrophy [42]; as such, this preliminary result may be worth following up. We also note that power to detect genuine genotype × sex interaction effects on hormone levels, analogous to those observed on bodyweight and anxiety‐related measures, was limited by small sample sizes, particularly of the homozygous female group. In STS‐deficient 39,X^Y^*O mice, we previously found no evidence for large changes in levels of brain steroids [43]; however, these mice did exhibit significantly lower levels of serum DHEA compared to 40,XY mice [14]. In the present analysis, median serum DHEA levels were lower, though not significantly so, in homozygous animals than in wildtype animals across both sexes. Future work in this model might examine levels of sulfated steroids, notably DHEAS and cholesterol sulfate; these compounds have multiple physiological roles, and accumulation of the latter is thought to underlie the skin pathology associated with XLI [44]. Potentially, the accumulation of steroid sulfates, rather than reduced levels of free steroids, might explain some of the phenotypes we have observed.

Finally, we have shown that STS‐deficient mice possess heavier hearts (normalised to bodyweight/tibia length) than their wildtype counterparts. Follow‐up work will aim to understand the basis and sequelae of this result: structural analyses will test whether there is any evidence of predicted hypertrophy, while functional analyses will test for any associated effects on electrical signalling using electrocardiography (ECG). Our ongoing ex vivo ECG studies indicate that hearts from STS‐deficient mice exhibit more frequent and more persistent ventricular arrhythmias than those from wildtype mice, and we suspect that prolonged exposure to such episodes may ultimately result in cardiac hypertrophy, as has been shown in other animal models [45]. Analyses in the skin of STS‐deficient mice have demonstrated altered Hippo signalling and elevated levels of phosphorylated Yes‐associated Protein 1 (YAP1) [23]. In cardiac cell culture, hyperactivation of YAP1 is associated with excessive Cellular Communication Network Factor 2 (CCN2) and Transforming Growth Factor‐beta (TGFβ) secretion and hypertrophy [46]. Expression of these biochemical markers in STS‐deficient mouse hearts may be perturbed, and warrants investigation. As cardiac screening in individuals with XLI becomes more commonly implemented, it will be interesting to see whether there is any evidence for hypertrophy in this group.

## 5 Conclusion

We have successfully generated a novel mouse model lacking STS activity, which exhibits normal breeding performance and is grossly healthy. The model displays behavioural and cardiac phenotypes of relevance to XLI, but no large changes in serum steroid hormone biochemistry. Future work should aim to replicate the initial significant findings reported here, and to investigate the anatomical, cellular, and molecular mechanisms underlying any robust results. More sophisticated phenotypic analyses, including of cognitive and cardiac function, might also be undertaken.

## Funding

Work was funded by an MRC GW4 DTP PhD studentship to Freya Shepherd, by a Cardiff University Innovation Development Scheme studentship to Talia Elgie, and by Cardiff University Schools of Psychology, Biosciences and Medicine. The funders played no role in the design, analysis, or reporting of the study.

## Ethics Statement

Experiments were performed according to the UK Animal Scientific Procedures Act (1986) under Home Office Project Licences P825DDA5B, PP2050169 and PP6583178 and reporting complies with the ARRIVE guidelines.

## Conflicts of Interest

The authors declare no conflicts of interest.

## Supporting information


**Figure S1:** gbb70061‐sup‐0001‐Supinfo.docx. *Sts* gene expression patterns in liver tissue of wildtype (+/+) and homozygous (−/−) male and female mice.
**Figure S2:** Performance of adult male and female wildtype (+/+) and homozygous (−/−) mice on key measures of consummatory behaviour (volume consumed per bodyweight (A) and preference for milk over water (B)) on the milk preference test.
**Figure S3:** Performance of adult male and female wildtype (+/+) and homozygous (−/−) mice on the rotarod test (median time to fall from an increasingly rapidly rotating rod across 5 individual trials).
**Figure S4:** Performance of adult male and female wildtype (+/+) and homozygous (−/−) mice on the startle (A) and prepulse inhibition (B) test. Prepulses of 4, 8 and 16 dB were used (PP4, PP8 and PP16 respectively).

**Supporting Information: S1.** Raw data for wildtype and homozygous deletion mice.

**Supporting Information: S2.** Raw behavioural data for heterozygous deletion mice.

## Data Availability

Raw data are available within this manuscript or within the Supporting Information [Link], [Link], [Link] files.
